# Prevalence and Risk Factors of MASLD and Liver Fibrosis amongst the Penitentiary Population in Catalonia: The PRISONAFLD Study

**DOI:** 10.3390/jcm12237276

**Published:** 2023-11-24

**Authors:** Jesús Rivera-Esteban, Alba Jiménez-Masip, Sergio Muñoz-Martínez, Salvador Augustin, Rafael A. Guerrero, Pablo Gabriel-Medina, Roser Ferrer-Costa, Francisco Rodríguez-Frías, Elisabet Turu, Andrés Marco, Juan M. Pericàs, on behalf of the PRISONAFLD Study Group Collaborators

**Affiliations:** 1 Liver Unit, Vall d’Hebron Institut de Recerca (VHIR), Vall d’Hebron Hospital Universitari, Vall d’Hebron Barcelona Hospital Campus, 08035 Barcelona, Spain; albamaria.jimenez@vallhebron.cat (A.J.-M.); sergio.munoz@vhir.org (S.M.-M.); salvador.augustin@boehringer-ingelheim.com (S.A.); 2 Department of Medicine, Universitat Autònoma de Barcelona, 08193 Barcelona, Spain; pablo.gabriel@vallhebron.cat (P.G.-M.); roser.ferrer@vallhebron.cat (R.F.-C.); francisco.rodriguez@vhir.org (F.R.-F.); 3 Prison Health Program, Catalan Institute of Health, 08908 Barcelona, Spain; rguerrero@gencat.cat (R.A.G.); eturu@gencat.cat (E.T.); amarco@gencat.cat (A.M.); 4 Clinical Biochemistry Department, Vall d’Hebron Hospital Universitari, 08035 Barcelona, Spain; 5 Ciber de Epidemiología y Salud Pública (CIBERESP), 28029 Madrid, Spain; 6 Centros de Investigación Biomédica en Red, Enfermedades Hepáticas y Digestivas (CIBERehd), 28029 Madrid, Spain

**Keywords:** non-alcoholic fatty liver disease, metabolic dysfunction-associated steatotic liver disease, metabolic syndrome, liver fibrosis, prison

## Abstract

Background and Aims: The prevalence of chronic non-communicable diseases, particularly metabolic syndrome (MetS), has increased among the prison population. Nevertheless, we have limited data on metabolic dysfunction-associated steatotic liver disease (MASLD), the hepatic manifestation of this syndrome. We aimed to investigate the prevalence and risk factors of MASLD and MASLD-associated liver fibrosis in the penitentiary population in Catalonia, Spain. Method: A cross-sectional observational study involving eight penitentiary centers. Participants had at least one metabolic disorder and were at a closed-regimen penitentiary. Individuals with concomitant liver diseases and/or alcohol risk consumption were excluded. Significant fibrosis and MASLD were defined as liver stiffness ≥8 kPa and a controlled attenuation parameter ≥275 dB/m by vibration-controlled transient elastography (VCTE), respectively. After exclusions, metabolic inmates with VCTE were analyzed. Logistic regression analysis was performed to identify predictors of MASLD and MASLD-associated significant fibrosis. Results: Out of the 4338 inmates studied, 1290 (29.7%) had metabolic disorders, and 646 (14.9%) underwent VCTE. The mean age was 48.0 years (SD 12.1), and 89.5% were male. MASLD prevalence was 33.9%. Significant fibrosis and MASLD-associated significant fibrosis were found in 16.4% and 9.4% of inmates, respectively. In the multivariate analysis, T2D, waist circumference, MetS, and higher ALT values were identified as independent risk factors for MASLD and MASLD-associated significant fibrosis amongst the prison population. Conclusions: Metabolic disorders including MASLD are highly prevalent among inmates. The prevalence of significant fibrosis seems notably higher than that of the general population, underscoring the need for targeted screening programs and therapeutic interventions in the incarcerated population.

## 1. Introduction

In recent decades, the occurrence of non-communicable chronic diseases has dramatically increased in the general population worldwide. This rise has been linked to the aging of the population and unhealthy lifestyles, including poor nutritional habits and reduced physical activity [1]. 

Metabolic syndrome (MetS) represents the quintessence of non-communicable chronic diseases. MetS encompasses multiple highly prevalent disorders, such as diabetes mellitus, obesity, high blood pressure, and dyslipidemia [2]. These conditions play a pivotal role in the development of neoplasms and cardiovascular, respiratory, and hepatic diseases, which are the main causes of death in developed countries [3]. The impact of MetS on morbidity and life expectancy explains why it is acknowledged as a major global health concern and a high-priority objective on the international health agenda [4].

Non-alcoholic fatty liver disease (NAFLD) or metabolic dysfunction-associated steatotic liver disease (MASLD), as recently renamed by a consensus panel of global experts [5], represents the hepatic manifestation of MetS. MASLD is characterized by hepatic fat accumulation in the absence of deleterious alcohol consumption, and it is strongly related to obesity and insulin resistance. MASLD is a progressive condition that can lead to inflammation, liver fibrosis, and even cirrhosis. The development of liver fibrosis is a milestone in the natural course of the disease, as it remarkably increases the risk of both liver and non-liver-related events and all-cause mortality [6,7,8].

Population-based studies in our setting indicate a prevalence of approximately 30% for MetS and 25–30% for MASLD [8,9]. Furthermore, a recent observational study including histology data suggested that about 2.03% of the general population in Spain would present significant fibrosis and 0.7% would have cirrhosis due to MASLD [10].

The dramatic increase in the prevalence and impact of MASLD has led to the development and validation of non-invasive diagnostic tests (NITs). Serum-based NITs, such as the FIB-4 index, are employed as the initial screening tool for liver fibrosis assessment in both the general population and high-risk groups [11,12]. Vibration-controlled transient elastography (VCTE) provides an accurate estimation of the degree of liver fibrosis and enables the detection of hepatic steatosis through the controlled attenuation parameter (CAP). VCTE has been used in several population-based studies and is broadly implemented in clinical practice [12,13,14]. 

The prison population is influenced by the same evolving lifestyle patterns and global health trends as the general population, and, additionally, exhibits distinct characteristics that render it an especially vulnerable demographic with a greater susceptibility to illness. There are several reasons for this increased predisposition, including unfavorable social determinants, as many prisoners originate from the third or fourth world, a higher incidence of mental disorders, and their engagement in high-risk behaviors and lifestyles, such as drug abuse [15,16]. 

In recent years, chronic non-communicable diseases have surpassed infectious diseases as the leading cause of death in prisons. This shift is mostly driven by cardiovascular events, secondary to progressive aging and the growing burden of metabolic disorders in the incarcerated population [17,18,19,20]. Notwithstanding these worrisome data, prior studies on the penitentiary population did not comprehensively examine MetS, offering only partial insights and veiling the complete picture [21,22]. 

Regarding MASLD, some aspects of the penitentiary population would promote the onset and progression of the disease. It has been described that people with low socioeconomic status are more likely to develop MASLD, MASLD-associated fibrosis, and death [23]. In addition, specific ethnicities overrepresented in prisons compared to the general population (i.e., Hispanics) have been associated with increased genetic susceptibility to MASLD, frequently attributed to single nucleotide polymorphisms (SNPs) in well-established genes, including PNPLA3 (patatin-like phospholipase domain containing 3) and TM6SF2 (transmembrane 6 superfamily member 2) [24].

Previous studies assessing liver diseases within the prison population left relevant issues unresolved. The research focus on liver disease in this population was placed on viral hepatitis and alcohol abuse [25,26]; the deployment of VCTE in this setting was uncommon [27], and no dedicated strategies for MASLD screening have been evaluated so far amongst prisoners [28,29]. Consequently, epidemiological data on metabolic disorders, MASLD, and MASLD-related liver fibrosis are scarce, and the optimal diagnostic approach in this population has yet to be established.

The present study aimed to describe the prevalence of MASLD and to identify predictors of MASLD and MASLD-associated liver fibrosis amongst the prison population in Catalonia, Spain. Additionally, we assessed the diagnostic performance of serum-based NITs for MASLD screening among inmates. Finally, considering the increased ethnic diversity in prisons compared to the Spanish general population, we evaluated the influence of SNPs for PNPLA3 and TM6SF2 genes in the development of MASLD and liver fibrosis within this population. 

## 2. Material and Methods

### 2.1. Patients and Setting

This was an observational, cross-sectional, multicentric study involving the penitentiary population of Catalonia, Spain. Study procedures and data collection were carried out from October 2021 to March 2022.

Catalan prisons are administered through a specific program that depends on the autonomous community government. This platform included a prison health program linked to tertiary public hospitals. The eight prisons in Catalonia housing inmates in a closed regime participated in the study, entailing a targeted population of 4338 inmates. These prisons were: (1) Brians-1 Women’s Department (Barcelona); (2) Brians-2 penitentiary center (Barcelona); (3) La Roca-1 penitentiary center (Quatre Camins, Barcelona); (4) La Roca-2 penitentiary center (Quatre Camins, Barcelona); (5) Lledoners penitentiary center (Barcelona); (6) Ponent penitentiary center (Lleida); (7) Mas d’Enric penitentiary center (Tarragona); and (8) Puig de les Basses penitentiary center (Figueres, Girona).

The study was designed and performed following the STROBE guidelines [30]. 

### 2.2. Inclusion Criteria 

We included inmates who met the following criteria: (1) >18 years old; (2) a closed imprisonment regime at the moment of the study, meaning that inmates had already been judged and placed in a specific prison with restricted mobility; (3) the presence of at least one metabolic disorder defined as (a) obesity: body mass index (BMI) ≥30 kg/m^2^ and/or waist circumference >102 cm for men and >88 cm for women, respectively; (b) high blood pressure (HBP): ≥130/85 mmHg or requiring treatment; (c) type 2 diabetes (T2D): fasting plasma glucose ≥126 mg/dL or a glycated hemoglobin ≥6.5% or requiring treatment; (d) dyslipidemia: serum triglycerides ≥150 mg/dL and/or total cholesterol >200 mg/dL, LDL >130 mg/dL, HDL < 40 mg/dL in men and <50 mg/dL in women or requiring treatment.

### 2.3. Exclusion Criteria 

The exclusion criteria were the following: (1) presence of hepatitis B surface antigen, hepatitis C virus (HCV) RNA, HCV-related cirrhosis or recent HCV treatment with direct-acting antiviral agents (<3 years from study initiation), and other causes of chronic liver disease (e.g., autoimmune diseases); (2) alcohol risk consumption (>30 gr/day in males and >20 gr/day in females) or AUDIT test ≥8 points in order to exclude those patients with high-risk of alcohol-related liver disease [5]; (3) patient’s release or inability to participate.

### 2.4. Outcomes

We aimed to describe MASLD prevalence and severity among inmates and to identify predictors of MASLD and MASLD-associated liver fibrosis. 

As an exploratory analysis, we examined the performance of serum-based NITs for MASLD detection and the impact of PNPLA3 and TM6SF2 SNPs in the development of MASLD and liver fibrosis in this population.

### 2.5. Definitions

MASLD was defined as the presence of steatosis assessed by VCTE-CAP ≥275 dB/m [12] in patients with at least one metabolic disorder and no other liver etiologies. 

Liver stiffness (LS) measurements ≥8 kPa by VCTE were considered suggestive of significant fibrosis [12,13,14]. Likely compensated advanced chronic liver disease (cACLD) was defined as LS ≥ 15 kPa, and the “rule of five” was applied for fibrosis distribution according to the latest Baveno consensus criteria [31]. 

Metabolic syndrome was defined according to NCEP-ATP-III criteria [32]. 

Serum NITs encompassed the FIB-4 index, Fatty Liver Index (FLI), and Enhanced Liver Fibrosis (ELF) score. A FIB-4 threshold ≥1.3/2.0 according to age (younger or older than 65, respectively) and ELF score ≥ 9.8 classified patients as high-risk for liver fibrosis. A FLI >60 enables the detection of steatosis and identifies patients at risk for significant fibrosis [33].

rs738409 CG/GG alleles from PNPLA3 gene and rs58542926 TC/TT alleles from TM6SF2 were considered high-risk haplotypes [24].

### 2.6. Procedures

During the study visit, we collected epidemiological variables, alcohol consumption data, and anthropometric data by physical examination. On the same day, fasting blood samples were obtained, including viral hepatitis and HIV serologies, and the AUDIT test was performed. Inmates with an AUDIT score of <8 points underwent a VCTE examination (Fibroscan 530 Compact, Echosens, France) at that time, conducted by experienced operators using the M and XL probes based on device recommendations [34]. The CAP algorithm is incorporated into VCTE devices and automatically calculates the attenuation of the ultrasound signal during the VCTE examination, correlating with the histological degree of steatosis [12,13,14].

The techniques employed in our study were selected based on the recommendations of international societies for MASLD and cACLD evaluation [13,31]. Serum NITs (FIB-4 index, FLI, and ELF) are commonly used as the first tests to identify patients at risk of liver fibrosis [11,33]. VCTE and CAP are the most validated NITs for the estimation of liver stiffness and steatosis, respectively [12,13,14]. Finally, PNPLA3 and TM6SF2 genes have been associated with a higher risk of liver fibrosis, hepatocellular carcinoma, and cardiometabolic events in patients with liver disease and MASLD [24]. 

VCTE quality criteria, ELF, and genetic analysis details are provided in the Supplementary Material.

### 2.7. Statistical Analysis 

Group comparisons were conducted using the χ^2^ test for qualitative variables, while for quantitative variables, student-t and non-parametric tests were applied, depending on whether they followed a normal or non-normal distribution, respectively. Frequencies and percentages were used to present categorical data, whereas continuous data were represented as either the mean ± standard deviation or the median (interquartile range) when it was suitable. Missing values were kept as missing, and no dedicated statistical analyses were employed for imputations.

Receiver operating characteristic (ROC) curves of serum-based NITs were developed for MASLD and MASLD-associated fibrosis detection. A logistic regression analysis was performed to identify risk factors of MASLD and MASLD-associated fibrosis. Only variables with *p* < 0.1 in univariate analysis were included for multivariate modeling. 

A *p* < 0.05 was considered statistically significant. Data were collected and edited using Microsoft Excel (version 2310; Microsoft Office Pro 2019). PAWS Statistics (version 19.0; SPSS Inc., Hong Kong) software was employed for statistical analyses. 

## 3. Results

### 3.1. Sample

**Figure 1:**
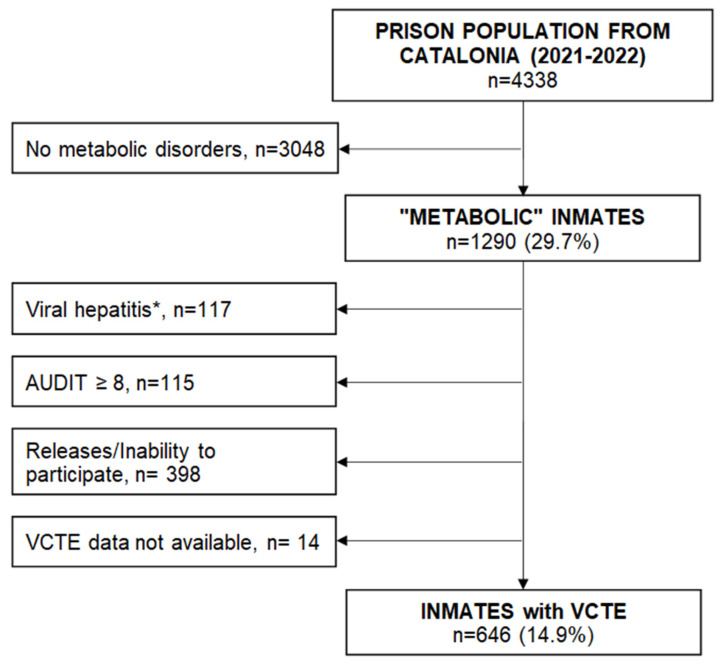
Flowchart of the study. * Viral hepatitis: Presence of hepatitis B surface antigen, positive hepatitis C virus (HCV) RNA, HCV-related cirrhosis, or recent HCV treatment with direct action antivirals (<3 years). VCTE; vibration-controlled transient elastography; AUDIT, Alcohol Use Disorders Identification Test.

Of the 4338 inmates evaluated, 1290 (29.7%) presented at least one metabolic disorder. Losses counted for 398 inmates, mainly due to inability to participate or prison release during the study period. After exclusions due to viral hepatitis and alcohol risk consumption, 646 patients entered the final analysis (Figure 1).

### 3.2. Study Population

The median age was 48.0 years (SD 12.1), and only 62 (9.6%) were ≥65 years old. Most inmates were male (89.5%), and 36.5% were born in non-European countries. The prevalence of obesity and T2D was 37.4% and 18.5%, whilst 40.2% and 43.2% of the inmates presented high blood pressure and dyslipidemia, respectively. Out of the 646 inmates, at least 1 had a metabolic disorder, and 196 (31.9%) met the metabolic syndrome criteria. A diagnosis of HIV infection was established in 6.1% of the inmates, and 67 (10.3%) had HCV antibodies. 

Regarding hepatic parameters, the mean ALT and AST values were 22 U/L and 24 U/L, respectively. The MASLD prevalence was 33.9%. The mean LS was 6.5 kPa, and 106 (16.4%) inmates presented an LS ≥ 8 kPa.

When comparing prisoners with and without MASLD, MASLD inmates were older (49.5 vs. 47.3 years; *p* < 0.027), presented higher rates of obesity (57.1% vs. 36.1%; *p* < 0.001), T2D (28.8% vs. 18.0%; *p* < 0.002), MetS (44.0% vs. 25.6%; *p* < 0.001), and HIV (9.2% vs. 4.5%; *p* < 0.029). ALT, AST, and GGT values were superior amongst MASLD subjects, as were mean LS (7.9 vs. 5.6 kPa; *p* < 0.001) and FLI (71 vs. 54; *p* < 0.001) values (Table 1). Supplementary Table S1 shows the characteristics of the inmates with MetS according to the presence of MASLD.

### 3.3. Liver Fibrosis Distribution and Relationship with MASLD 

The prevalence of significant fibrosis estimated by VCTE in the whole cohort was 16.4%, while the proportion of patients with significant fibrosis due to MASLD was 9.4% (61/646). Supplementary Table S2 presents the characteristics of the inmates according to the LS 8 kPa cut-off, showing no differences regarding HCV serology. 

**Figure 2:**
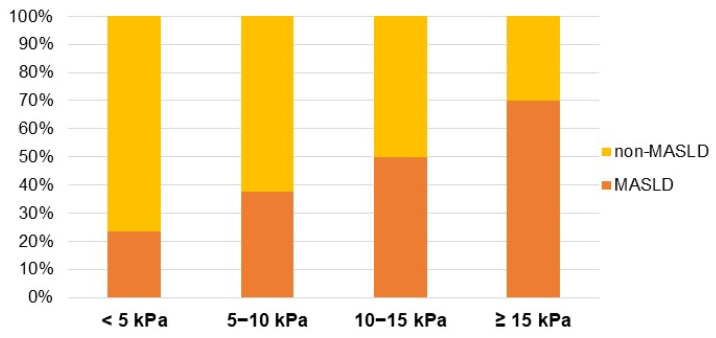
Distribution of the patients according to VCTE ranges and MASLD prevalence.

The distribution of patients applying the “rule of five” from the Baveno consensus is presented in Table 2 and depicted in Figure 2. The association of MASLD and liver fibrosis increased along LS values, ranging from 24% in the lowest LS interval to 70% in the subset of patients with cACLD (LS ≥ 15 kPa). 

Regarding serum-based NITs, the median FLI was significantly higher amongst those inmates with estimated significant fibrosis (71 ± 25 vs. 54 ± 26; *p* < 0.001), while no differences were found for FIB-4 index (1.02 ± 0.6 vs. 1.00 ± 0.5; *p* < 0.75) between prisoners with LS < 8 and ≥8 kPa.

The ELF score was performed on 238 inmates. The median ELF was 9.4 (IQR 8.8–9.8), and 75 (31.5%) presented an ELF score ≥9.8. The prevalence of metabolic disorders, including MASLD, tended to be higher among inmates with ELF ≥ 9.8, although no statistical differences were found (Supplementary Table S3).

Furthermore, we evaluated the diagnostic accuracy of the serum-based NITs using VCTE as the reference method. An FLI > 60 was found in 305 (51.7%) inmates, showing FLI an AUC of 0.69 (95%CI 0.64–0.73) for MASLD prediction. 

The AUC of FIB-4 index, FLI, and ELF score for MASLD-associated significant fibrosis detection were 0.48 (95%CI 0.33–0.63), 0.66 (95%CI 0.48–0.84), and 0.69 (95%CI 0.56–0.82), respectively. 

### 3.4. Predictors of MASLD and MASLD-Associated Liver Fibrosis among the Prison Population

In the multivariate regression analysis, metabolic disorders (T2D, increasing values of waist circumference), whether analyzed separately or within the metabolic syndrome, were identified as independent risk factors for both MASLD and MASLD-associated significant fibrosis, along with ALT values, in contrast to HIV infection (Table 3). 

### 3.5. Genetic Single Nucleotide Polymorphism Analysis

Out of the 79 (12.2%) inmates studied, 34 (43.0%) and 8 (10.1%) had PNPLA3 and TM6SF2 high-risk alleles, while 4 inmates (5.1%) presented high-risk alleles for both genes. The comparison between inmates with and without risk haplotypes is shown in Supplementary Table S4. Of note, HIV prevalence was superior amongst patients with high-risk alleles for PNAPL3 (*p* < 0.033) and TM6SF2 (*p* < 0.022). Finally, no statistical differences were observed between groups for the origin, the presence of metabolic disorders or MASLD, mean LS, or serum-based NIT values. 

## 4. Discussion

In the present study, we investigated the existence of MASLD and its association with liver fibrosis in prisoners with metabolic disorders in Catalonia. We found that a substantial proportion of inmates had suspected MASLD and significant fibrosis (34% and 16%, respectively). Metabolic disorders, such as T2D or obesity (estimated by waist circumference), and higher ALT values were identified as independent risk factors for MASLD and MASLD-associated significant fibrosis amongst the prison population. 

Historically, infectious diseases caused the bulk of hepatic and extrahepatic morbidity in the penitentiary setting, notably, viral hepatitis and HIV [18,19,20]. Nonetheless, in recent years, chronic non-communicable diseases have become the leading cause of death in prisons, mainly driven by cardiovascular and metabolic-related events, which are significantly more frequent in patients with concomitant advanced liver disease due to MASLD [35]. Our results confirmed the impact of metabolic disorders in the penitentiary population [36], putting the spotlight on the need for screening programs and targeted therapeutic interventions and reinforcing the role of abdominal circumference instead of body mass index as a marker of adiposity [37].

Despite the increasing awareness and the important health burden of MASLD, to the best of our knowledge, this is the first study that specifically addressed this disease in the penitentiary population. We found that a third of the inmates presented MASLD, and that weight as an etiology was progressively more prominent as fibrosis increased. In our cohort, applying the foreknown “rule of five” [31], we found that up to 70% of patients with suspected cACLD presented MASLD. Overall, these results underlie the impact of metabolic disorders on MASLD onset and progression and emphasize the role of MASLD as a major cause of liver fibrosis amongst the penitentiary population. Furthermore, it is worth highlighting that, despite caveats regarding the performance of VCTE in obese individuals, VCTE-based prediction models for liver-related events have been recently validated on MASLD patients, including those with obesity, with proper accuracy [38].

Beyond the direct impact of HIV on the health status of prisoners, we found a higher HIV prevalence in MASLD inmates and those patients with estimated significant fibrosis, although no statistical association was found in the regression analysis. This issue could be partially explained due to the relatively low number of HIV subjects and the median age of our cohort. It should be noticed that MASLD evolves slowly, and significant fibrosis typically emerges in patients older than 50 years. In any case, our results are aligned with previous findings, suggesting a close relationship between HIV, MASLD, and chronic liver disease [39,40,41]. Larger epidemiological studies with a broader range of ages are needed to confirm the role of HIV in the prison population with non-viral liver disease.

In the field of MASLD and liver fibrosis, refining and validating diagnostic pathways based on serum-based NITs is one of the contemporary foremost challenges, particularly in the non-tertiary setting, such as prisons, where VCTE availability is limited [13,42,43]. In our cohort, the performance of the FIB-4 index was suboptimal, probably due to the age distribution of our cohort. On the contrary, biomarkers based on metabolic parameters (i.e., FLI) or metabolites derived from the extracellular matrix (i.e., ELF score) could be a suitable option for fibrosis screening in the absence of VCTE devices [11,33]. Additional studies are required to evaluate the prognostic ability of NITs as well as the role of genetics and epigenetics in establishing the optimal diagnostic algorithm in this specific high-risk population.

The main strengths of the study are the large sample size analyzed, the number of VCTEs performed, and the greater ethnic and geographical diversity in our cohort compared to the general population of Spain. It is important to underline that our study was designed and performed during the COVID-19 pandemic and represents the largest effort in the evaluation of MetS, MASLD, and MASLD-associated fibrosis in the prison population to date. Moreover, we provided a complementary analysis of serum-based NITs, which are the first diagnostic tools used for MASLD and fibrosis screening in clinical practice outside liver clinics. 

The main limitations encompassed a low proportion of females, which is a common trait in the prison population, as consistently shown in previous reports in this setting [28,29]. Additionally, the number of non-Caucasian participants was lower than expected, although their representation notably exceeded that of the general population from Spain [10], limiting the analysis of the ethnic impact of SNPs within our cohort. These factors might restrict the applicability of our findings, underscoring the need for further research in more ethnically diverse populations. 

Besides, it is worth noting that fibrosis estimations and MASLD causality were not histologically back-tested, and results on serum-based NITs and SNPs should be interpreted with caution since genetic and ELF score analyses were performed on a relatively low number of subjects. The etiology attribution made in our study was imperfect, particularly in the non-MASLD group, since histological data were lacking. Burn-out MASLD inmates could have been misclassified based on lower CAP values, although the number of patients with suspected cACLD in this group is negligible. Additionally, no biological testing was performed to confirm alcohol abstinence, and genetic-based liver diseases or inherited abnormalities of metabolism were not routinely excluded, although the estimated prevalence is <2% in the general population. Finally, we did not gather data on metabolic disorders or HIV medications, precluding the analysis of the impact of antivirals or weight-loss drugs on MASLD and MASLD-fibrosis.

In conclusion, the penitentiary population appears to have a high prevalence of metabolic disorders including MASLD. T2D, obesity, MetS, and increasing ALT values were independently associated with MASLD and MASLD-associated liver fibrosis. 

The present study could serve as a benchmark for developing tailored metabolic and MASLD-dedicated screening strategies using parallel or sequential combinations of serum-based NITs to identify at-risk patients in the absence of VCTE devices within the penitentiary population. 

We believe this study paves the way for future longitudinal investigations that would specifically address high-risk groups, such as women, inmates living with HIV, and patients who develop MASLD following a cure or control of previous viral hepatitis, providing insights into the understanding and progression of this overlapping liver disease.

Finally, our findings hold significant implications for the design and implementation of interventions aimed at mitigating the disease burden associated with metabolic, cardiovascular, and hepatic conditions among inmates. Such interventions could encompass comprehensive lifestyle and dietary modifications, thereby contributing to improved health outcomes and quality of life for the incarcerated population.

## Figures and Tables

**Table 1:** Baseline characteristics of the population included in the study.

Variables	Overall *n* = 646	MASLD *n* = 219	Non-MASLD *n* = 427	*p*-Value
Age (years)	48.0 ± 12.1	49.5 ± 12.1	47.3 ± 12.1	0.027
Male sex, *n* (%)	578 (89.5)	198 (90.4)	380 (89.0)	0.57
Origin, *n* (%)				
Europe	410 (63.5)	137 (62.6)	273 (63.9)	0.73
Latin-America	123 (19.0)	39 (17.8)	84 (19.7)	0.56
Maghreb-Arab	66 (10.2)	26 (11.9)	40 (9.4)	0.32
Sub-Saharan Africa	23 (3.6)	6 (2.7)	17 (4.0)	0.42
Asia	26 (4.0)	11 (5.0)	15 (3.5)	0.35
Body mass index (kg/m^2^)	28.3 (25.6–31.7)	30.4 (27.0–32.5)	28.3 (25.1–31.2)	<0.001
Obesity (≥30 kg/m^2^), *n* (%)	239 (38.4)	117 (57.1)	151 (36.1)	<0.001
Waist circumference (cm)	100 (92–109)	106 (97–115)	97 (90–106)	<0.001
Type 2 diabetes, *n* (%)	140 (21.7)	63 (28.8)	77 (18.0)	0.002
High blood pressure, *n* (%)	265 (41.0)	98 (44.7)	167 (39.1)	0.16
Dyslipidemia, *n* (%)	416 (64.5)	148 (67.6)	268 (62.9)	0.24
Metabolic syndrome, *n* (%)	196 (31.9)	92 (44.0)	104 (25.6)	<0.001
HIV, *n* (%)	34 (6.1)	17 (9.2)	17 (4.5)	0.029
AST (U/L)	22 ± 8	23 ± 9	21 ± 6	0.017
ALT (U/L)	24 ± 14	27 ± 16	22 ± 11	<0.001
AP (UI/L)	82 ± 25	84 ± 25	81 ± 25	0.27
GGT (UI/L)	32 ± 27	36 ± 30	30 ± 24	0.007
Fasting glucose (mg/dL)	105 ± 59	114 ± 58	100 ± 59	0.004
Cholesterol (mg/dL)	187 ± 44	190 ± 45	185 ± 43	0.16
Triglycerides (mg/dL)	147 ± 89	168 ± 109	136 ± 74	<0.001
Creatinine (mg/dL)	0.90 ± 0.21	0.91 ± 0.44	0.89 ± 0.17	0.19
Platelet count (10^9^/L)	250 ± 64	251 ± 62	249 ± 65	0.79
Liver stiffness (kPa)	6.4 ± 5.6	7.9 ± 8.7	5.7 ± 2.5	<0.001
XL probe, *n* (%)	325 (50.3)	159 (72.6)	166 (38.9)	<0.001
CAP (dB/m)	253 ± 63	324 ± 37	216 ± 36	<0.001
FIB-4 index	1.00 ± 0.55	1.00 ± 0.54	1.00 ± 0.55	0.99
Fatty Liver Index	59 ± 26	71 ± 25	54 ± 26	<0.001
ELF score *	9.3 ± 0.8	9.4 ± 0.8	9.3 ± 0.8	0.44
PNPLA3 risk alleles **	34 (43.0)	14 (50.0)	20 (39.2)	0.35
TM6SF2 risk alleles **	8 (10.1)	2 (7.1)	6 (11.8)	0.51

* Data from 238 inmates. ** Data from 79 inmates. Abbreviations: CAP, controlled attenuation parameter; HIV, human immunodeficiency virus; ELF, Enhanced Liver Fibrosis. Qualitative variables were analyzed by χ^2^ and expressed as *n* (%). Student-*t* test was applied for quantitative variables with normal distribution and presented as mean ± standard deviation, while non-normal quantitative variables were assessed by the Mann–Whitney U test and represented as median (interquartile range).

**Table 2:** Distribution of the patients according to vibration-controlled transient elastography ranges.

VCTE Ranges	MASLD	Non-MASLD	Total
<5 kPa, *n* (%)	58 (26.4)	188 (44.0)	246 (38.1)
5–10 kPa, *n* (%)	131 (59.8)	217 (50.7)	348 (53.9)
10–15 kPa, *n* (%)	16 (7.4)	16 (3.7)	32 (4.9)
≥15 kPa, *n* (%)	14 (6.4)	7 (1.6)	20 (3.1)
Total	219 (33.9)	427 (66.1)	646 (100)

**Table 3:** Predictors of MASLD and MASLD-associated significant fibrosis by vibration-controlled transient elastography.

Variables	MASLD		MASLD-Significant Fibrosis	
	OR (95%CI)	*p*-Value	OR (95%CI)	*p*-Value
Model 1				
T2D	1.70 (1.08–2.67)	0.02	1.99 (1.00–3.99)	0.050
Waist circumference *	2.91 (1.97–4.30)	<0.001	3.58 (1.74–7.31)	0.001
ALT (40 UI/L)	2.42 (1.30–4.49)	0.005	4.06 (1.87–8.80)	<0.001
HIV infection	1.18 (0.51–2.72)	0.69	2.02 (0.67–3.04)	0.20
Model 2				
MetS	2.18 (1.47–3.22)	<0.001	2.14 (1.14–4.03)	0.018
ALT (40 UI/L)	2.52 (1.35–4.47)	0.003	4.20 (1.98–8.89)	<0.001
HIV infection	1.67 (0.76–3.62)	0.19	2.25 (0.79–6.44)	0.12

* Waist circumference >102 cm in men and >88 cm in women.

## Data Availability

Data available on request due to privacy restrictions. The data presented in this study are available on request from the corresponding author. The data are not publicly available due to privacy restrictions.

## Supplementary Materials

The following supporting information can be downloaded at: https://www.mdpi.com/article/10.3390/jcm12237276/s1, Table S1: Baseline characteristics according to the presence of MASLD amongst inmates with metabolic syndrome; Table S2: Baseline characteristics according to the presence of estimated significant fibrosis by vibration-controlled transient elastography; Table S3: Baseline characteristics according to the ELF score threshold; Table S4: Baseline characteristics according to the presence of high-risk alleles of PNPLA3 and TM6SF2 genes.

## Abbreviations

BMI, Body mass index; MASLD, Metabolic dysfunction-associated steatotic liver disease; CAP, Controlled Attenuation Parameter; VCTE, Vibration-controlled transient elastography; HBP, high blood pressure; HDL, high-density lipoprotein; LDL, low-density lipoprotein; ALT, alanine aminotransferase; AST, aspartate aminotransferase; ALP, alkaline phosphatase; GGT, gamma-glutamyl transferase; CI, confidence interval; T2D, type 2 diabetes; ELF, Enhanced Liver Fibrosis.
