# Supplementary material for: Prevalence and Risk Factors of MASLD and Liver Fibrosis amongst the Penitentiary Population in Catalonia: The PRISONAFLD Study
Source: J Clin Med. 2023 Nov 24;12(23):7276. doi: 10.3390/jcm12237276 (PMC10707608; doi:10.3390/jcm12237276)
Supplement: Supplementary file 1 [file jcm-12-07276-s001.zip › jcm-2681430-supplementary.pdf]

Supplementary Procedures:

VCTE quality criteria: We considered reliable those VCTE explorations with an interquartile range/median (IQR/M) ratio <0.30 and at least 10 valid individual measurements.

ELF score: We measured hyaluronic acid (HA), procollagen III N-terminal propeptide (PIIINP), and tissue inhibitor of matrix metalloprotease-1 (TIMP-1) and the ELF score using an Advia Centaur XP (Siemens Healthcare, Erlangen, Germany).

Genetic analysis: DNA was extracted from serum samples by the MagNa Pure 24 system. The *rs738409 PNPLA3* and *rs58542926 TM6SF2* SNPs were assessed by allele-specific genotyping techniques with real-time polymerase chain reaction (qPCRr) and fluorescent resonance energy transfer (FRET) specific probes and melting peaks analysis (Light SNiP assay) on a capillary-based thermocycler.

Supplementary Table S1. Baseline characteristics according to the presence of MASLD amongst inmates with metabolic syndrome.

**Variables**	Overall MetS N=196	MASLD N=97	Non-MASLD N=104	p-value
Age (years)	52.0±11.5	53.3±12.8	50.8±10.2	0.12
Male sex, n (%)	177 (90.3)	82 (89.1)	95 (91.3)	0.60
Origin, n (%)				
Europe	137 (69.9)	66 (71.7)	71 (68.3)	0.59
Latin-America	28 (14.3)	12 (13.0)	16 (15.4)	0.64
BMI (kg/m2)	29.2 (26.3-32.3)	29.6 (27.2-34.1)	28.9 (26.0-31.7)	0.08
Obesity, n (%)	99 (51.3)	51 (56.7)	48 (46.6)	0.16
Waist circumference (cm)	108 (100-114)	110 (103-120)	105 (98-111)	<0.001
T2D, n (%)	98 (50.0)	49 (53.3)	49 (47.1)	0.39
HBP, n (%)	130 (66.3)	64 (69.6)	66 (63.5)	0.36
Dyslipidemia, n (%)	123 (62.8)	65 (70.7)	58 (55.8)	0.031
HIV, n (%)	10 (6.1)	4 (5.3)	6 (6.7)	0.72
AST (U/L)	21±7	22±7	20±7	0.15
ALT (U/L)	24±13	25±13	23±11	0.19
AP (UI/L)	86±29	87±259	85±29	0.70
GGT (UI/L)	34±27	36±23	32±19	0.22
Fasting glucose (mg/dL)	125±64	139±77	112±44	0.004
Cholesterol (mg/dL)	181±46	188±51	174±41	0.035
Triglycerides (mg/dL)	197±113	222±135	176±84	0.007
Creatinine (mg/dL)	0.90±0.25	0.87±0.17	0.92±0.30	0.21
Platelet count (109/L)	258±69	261±64	255±72	0.57
Liver stiffness (kPa)	6.3±3.3	7.1±4.1	5.6±2.1	0.001
CAP (dB/m)	276±59	329±36	229±28	<0.001
FIB-4 index	1.00±0.57	1.00±0.52	1.00±0.61	0.91
Fatty Liver Index	75±19	81±17	70±21	<0.001
ELF score*	9.5±0.7	9.6±0.8	9.5±0.8	0.79
PNPLA3 risk alleles**	14 (53.8)	8 (57.1)	6 (50.0)	0.71
TM6SF2 risk alleles**	2 (7.7)	1 (7.1)	1 (8.3)	0.91

*Data from 67 inmates. **Data from 26 inmates. Qualitative variables were analyzed by χ2 and expressed as n (%). t-Student test was applied for quantitative variables with normal distribution and presented as mean ± standard deviation, whilst non-normal quantitative variables were assessed by Mann-Whitney U test and represented as median (interquartile range).

Abbreviations: BMI, Body mass index; CAP, controlled attenuation parameter; HBP, high blood pressure; HIV, human immunodeficiency virus; T2D, type 2 diabetes, ELF, Enhanced Liver Fibrosis.

Supplementary Table S2. Baseline characteristics according to the presence of estimated significant fibrosis by vibration-controlled transient elastography.

Variables	LS < 8 kPa<br>N=540	LS ≥ 8 kPa<br>N=106	p-value
Age (years)	47.8±12.2	49.1±11.6	0.32
Male sex, n (%)	480 (88.9)	98 (92.5)	0.27
Origin, n (%)			
Europe	336 (62.2)	74 (69.8)	0.13
Latin-American	107 (19.8)	16 (15.1)	0.25
BMI (kg/m^2)	28.0 (25.4-31.1)	30.0 (25.8-34.1)	0.005
Obesity (≥30 kg/m^2), n (%)	188 (36.1)	51 (50.0)	0.008
Waist circumference (cm)	99 (91-108)	105 (96-115)	0.001
T2D, n (%)	109 (20.2)	31 (29.2)	0.038
HBP, n (%)	219 (40.6)	46 (43.4)	0.58
Dyslipidemia, n (%)	345 (64.0)	71 (67.0)	0.55
MASLD, n (%)	158 (29.3)	61 (57.5)	<0.001
HIV, n (%)	23 (4.8)	11 (12.8)	0.004
HCV, n (%)	54 (10.9)	13 (13.0)	0.53
AST (U/L)	22±7	24±11	0.18
ALT (U/L)	23±13	27±18	0.052
GGT (UI/L)	31±23	41±38	0.008
Fasting glucose (mg/dL)	103±60	113±54	0.13
Cholesterol (mg/dL)	187±44	184±45	0.53
Triglycerides (mg/dL)	145±87	154±97	0.35
Creatinine (mg/dL)	0.90±0.44	0.90±0.26	0.96
Platelet count (10^9/L)	250±63	249±68	0.81
Liver stiffness (kPa)	5.1±1.23	13.1±11.3	<0.001
XL probe, n (%)	268 (49.6)	57 (53.8)	0.43
CAP (dB/m)	249±59	285±70	<0.001
FIB-4 index	1.00±0.54	1.02±0.60	0.75
Fatty Liver Index	58±26	67±27	0.002
ELF score*	9.3±0.8	9.9±0.8	0.002

PNPLA3 risk alleles**	27 (44.3)	7 (38.9)	0.68
TM6SF2 risk alleles**	6 (9.8)	2 (11.1)	0.87

*Data from 238 inmates. **Data from 79 inmates. Qualitative variables were analyzed by χ2 and expressed as n (%). t-Student test was applied for quantitative variables with normal distribution and presented as mean ± standard deviation, whilst non-normal quantitative variables were assessed by Mann-Whitney U test and represented as median (interquartile range).
Abbreviations: BMI, Body mass index; CAP, controlled attenuation parameter; HBP, high blood pressure; HIV, human immunodeficiency virus; HCV, Hepatitis C virus; T2D, type 2 diabetes, ELF, Enhanced Liver Fibrosis.

Supplementary Table S3. Baseline characteristics according to the ELF score threshold.

**Variables**	**ELF < 9.8** N=163	**ELF ≥ 9.8** N=75	**p-value**
Age (years)	43.9±11.1	51.4±11.8	<0.001
Male sex, n (%)	139 (85.3)	59 (78.7)	0.20
BMI (kg/m^2)	28.9±4.7	30.1±7.3	0.21
Obesity, n (%)	60 (36.8)	31 (41.3)	0.50
T2D, n (%)	32 (19.6)	22 (29.3)	0.09
HBP, n (%)	56 (34.4)	33 (44.0)	0.15
Dyslipidemia, n (%)	110 (67.5)	47 (62.7)	0.46
MASLD, n (%)	54 (33.1)	30 (40.0)	0.30
HIV, n (%)	7 (4.7)	6 (9.0)	0.21
AST (U/L)	22±7	22±11	0.70
ALT (U/L)	23±13	23±18	0.86
GGT (UI/L)	28±18	32±31	0.33
Glucose (mg/dL)	104±39	113±55	0.16
Cholesterol (mg/dL)	191±46	186±45	0.40
Triglycerides (mg/dL)	143±93	155±102	0.35
Platelet count (10^9/L)	250±64	250±75	0.96
Liver stiffness (kPa)	5.4±1.7	6.9±5.1	0.017
CAP (dB/m)	258±58	264±61	0.48
FIB-4 index	0.94±0.48	1.10±0.71	0.09
Fatty Liver Index	54±27	61±25	0.057

Qualitative variables were analyzed by χ2 and expressed as n (%). t-Student test was applied for quantitative variables with normal distribution and presented as mean ± standard deviation.
Abbreviations: BMI, Body mass index; CAP, controlled attenuation parameter; HBP, high blood pressure; HIV: human immunodeficiency virus; T2D, type 2 diabetes.

Supplementary Table S4. Baseline characteristics according to the presence of high-risk alleles of PNPLA3 and TM6SF2 genes.

Variables	PNPLA3			TM6SF2		
	**rs738409 CC** N=45	**rs738409 CG/GG** N=34	**p-value**	**rs58542926 CC** N=71	**rs58542926 TC/TT** N=8	**p-value**
Age (years)	48.1±13.6	48.5±12.1	0.89	49.2±12.8	40.7±11.8	0.09
Male sex, n (%)	42 (93.3)	30 (88.2)	0.43	64 (90.1)	8 (100)	0.35
Origin, n (%)						
Europe	27 (60.0)	16 (47.1)	0.25	40 (56.3)	3 (37.5)	0.31
Latin-America	7 (15.6)	11 (32.4)	0.07	15 (21.1)	3 (37.5)	0.29
BMI (kg/m^2)	28.5±5.4	29.0±4.3	0.60	28.8±5.0	27.6±4.0	0.52
Obesity, n (%)	12 (26.7)	15 (44.1)	0.10	26 (36.6)	1 (12.5)	0.17
T2D, n (%)	12 (26.7)	9 (26.5)	0.98	18 (25.4)	3 (37.5)	0.46
HBP, n (%)	17 (37.8)	16 (47.1)	0.40	29 (40.8)	4 (50)	0.61
Dyslipidemia, n (%)	26 (57.8)	23 (67.6)	0.37	46 (64.8)	3 (37.5)	0.13
MASLD, n (%)	14 (31.4)	14 (41.2)	0.35	26 (36.6)	2 (25)	0.51
HIV, n (%)	1 (2.4)	5 (16.7)	0.033	4 (6.2)	2 (33.3)	0.022
AST (U/L)	22±7	23±11	0.56	23±10	23±8	0.95
ALT (U/L)	25±15	27±14	0.49	26±15	23±12	0.60
GGT (UI/L)	36±36	31±17	0.49	35±31	26±6	0.33
Glucose (mg/dL)	99±23	100±32	0.82	98±19	112±66	0.57
Cholesterol (mg/dL)	186±49	183±37	0.75	186±45	171±25	0.33
Triglycerides (mg/dL)	126±72	153±87	0.13	138±83	128±42	0.73
Liver stiffness (kPa)	7.2±5.5	6.1±1.9	0.28	6.8±4.5	6.6±2.5	0.90
CAP (dB/m)	253±65	264±62	0.48	260±65	236±49	0.31
FIB-4 index	1.17±0.80	1.10±0.69	0.73	1.14±0.70	1.22±1.15	0.78
Fatty Liver Index	53±27	62±25	0.13	58±26	51±24	0.46

Qualitative variables were analyzed by $\chi 2$ and expressed as n (%). t-Student test was applied for quantitative variables with normal distribution and presented as mean ± standard deviation. Abbreviations: BMI, Body mass index; CAP, controlled attenuation parameter; HBP, high blood pressure; HIV: human immunodeficiency virus; T2D, type 2 diabetes.
